# Genome-Wide Identification and Characterization of NAC Transcription Factors in Avocado (*Persea americana*): Expression Analysis During Fruit Development

**DOI:** 10.3390/genes17060706

**Published:** 2026-06-18

**Authors:** Zhijiao Song, Chengxian Wang, Mingliang Zhang, Yu Zhao, Jiaxing Qi, Jingsong Guo, Zhicai Zhang, Qing Liu

**Affiliations:** School of Resources and Environment, Baoshan University, Baoshan 678000, China; wangcxv@163.com (C.W.); yxmingliang@126.com (M.Z.); z1029793273@163.com (Y.Z.); qijiaxing930127@163.com (J.Q.); bsxy10939@bsc.edu.cn (J.G.); 18187571570@163.com (Z.Z.)

**Keywords:** NAC transcription factors, *P. americana*, genome-wide identification, phylogenetic analysis, fruit development and ripening

## Abstract

**Background**: The NAC family constitutes one of the largest families of plant-specific transcription factors and plays crucial roles in fruit development, ripening, seed life, and stress responses. However, comprehensive characterization of *NAC* genes in *Persea americana* (avocado), an economically important horticultural crop, has been largely unexplored. **Methods**: We performed a genome-wide identification and systematic characterization of NAC transcription factor (TF) genes in *P. americana* using blastp analysis, phylogenetic reconstruction, expression profiling and weighted gene co-expression network analysis (WGCNA). **Results**: A total of 130 *NAC* genes (*PaNACs*) were identified and distributed across all 12 chromosomes. Phylogenetic analysis classified these *PaNACs* into eight distinct subfamilies. WGCNA identified 43 co-expression modules, with 68 *PaNAC* genes distributed across 24 modules associated with hormone signaling, cell wall modification, secondary metabolism, and fatty acid beta-oxidation. Among 48,785 developmental differentially expressed genes (DEGs), 70 *PaNAC* genes were differentially expressed, with *PaNAC003* and *PaNAC002* showing the strongest upregulation and *PaNAC023* and *PaNAC025* the strongest downregulation. Among 9488 ethylene-responsive DEGs, *PaNAC041* was suppressed by ethylene and induced by 1-methylcyclopropene (1-MCP, a competitive inhibitor of ethylene perception), while *PaNAC016*, *PaNAC085*, and *PaNAC086* showed the opposite pattern. **Conclusions**: These findings provide a genomic and transcriptional framework for future functional investigation of *PaNAC* genes and their potential relevance to avocado fruit development and postharvest ripening.

## 1. Introduction

*P*. *americana* (avocado) is a commercially important fruit crop valued for its nutritional quality, flavor, and expanding global market. As a climacteric fruit, avocado undergoes rapid physiological and biochemical transitions during ripening, including changes in texture, color, aroma, and respiratory activity [1]. These processes are tightly regulated by transcriptional reprogramming and hormonal control, especially through ethylene signaling [2]. The interplay between multiple phytohormones, including ethylene, abscisic acid, jasmonic acid, and gibberellins, in coordinating avocado ripening across different genotypes and environmental contexts has also been documented, with considerable variety- and location-dependent variation in the expression of ethylene biosynthesis and signaling genes [3,4]. Several transcription factor families have been characterized in avocado at the genome-wide level, including the fatty acid desaturase (FAD) gene family [5] and the auxin response factor (ARF) family [6], providing resources for understanding lipid metabolism and hormone signaling, respectively.

NAC TF, named after NAM, ATAF, and CUC, is a plant-specific family characterized by a conserved N-terminal DNA-binding NAC domain and a variable C-terminal transcriptional regulatory region [7,8]. The number of NAC genes identified in plants varies significantly, with 93 reported in tomato [9] and 488 in wheat [10] (nearly five times the number found in tomato). Members of this family have been implicated in a wide range of biological programs underlying plant growth and development processes, including embryogenesis [11], leaf senescence [12], fruit ripening [13,14,15], seed development [16,17], and root development [18]. In addition, NACs play central roles in plant responses to various biotic and abiotic stimuli, such as pathogen infection [19], drought [20], heat [21], cold [22], and salinity [23]. For instance, *MfNACsa*, a lipid-anchored NAC transcription factor, enhances drought tolerance by directly binding to the promoter of *MtGly1* to maintain glutathione redox homeostasis [20]. Under heat stress, overexpression of *ZmNAC074* in transgenic *Arabidopsis thaliana* confers thermotolerance by modulating ROS scavenging capacity and upregulating heat stress-responsive genes [21]. In the cold stress, *GmNAC20* promotes freezing tolerance by activating *DREB1A*/*CBF3* and *DREB1C*/*CBF2* expression, while *PbeNAC1* from pear enhances cold tolerance through interaction with DREB-family proteins to induce downstream stress-associated gene expression [22]. Particularly, some NACs have the capacity to alter ethylene production, including positive and negative regulation of ethylene biosynthesis gene expression [17]. For example, SlNAM1 in tomato can directly bind and activate the expression of *SlACS2* and *SlACS4*, thereby enhancing ethylene synthesis and fruit ripening [24]. AdNAC2 and AdNAC72 in kiwifruit can bind and activate the promoter of the methionine sulfoxide reductase gene *AdMsrB1*, thereby promoting ethylene production [25].

Despite the growing body of knowledge on NAC transcription factors in model species and other fruit crops, a systematic genome-wide characterization of *NAC* genes in avocado remains absent, even though avocado presents a distinct ripening physiology compared to well-studied climacteric fruits such as tomato and banana. Mesocarp oil accumulation reaches 15–30% of fresh weight and occurs primarily during on-tree maturation rather than postharvest ripening [26]. Fruit may remain on the tree for more than 12 months beyond physiological maturity without undergoing softening or visible color change, with harvest timing is determined by indirect maturity indices [27]. Furthermore, postharvest ethylene manipulation using 1-MCP produces maturity-stage-dependent effects on ripening progression, underscoring the sensitivity of avocado to ethylene signal disruption [28]. Therefore, characterizing the NAC transcription factor family in avocado and elucidating its dynamics during fruit ripening may offer new perspectives on the transcriptional mechanisms underlying this species’ distinctive maturation biology.

Here, we conducted a comprehensive genome-wide identification and characterization of NAC genes in avocado. We analyzed their phylogenetic relationships, chromosomal localization and structural features. In addition, we examined their expression patterns across multiple tissues, fruit developmental stages, and postharvest treatments with 1-methylcyclopropene (1-MCP) and ethephon. Furthermore, WGCNA was employed to identify NAC-containing modules associated with fruit maturation and construct subnetworks of key NAC candidates. This study provides a genomic and transcriptional framework for understanding *PaNAC* genes and for dissecting the role of *PaNAC* genes in fruit development and postharvest ripening in avocado.

## 2. Materials and Methods

### 2.1. Plant Materials and RNA Extraction and Sequencing

*P. americana* line of Hass were cultivated at Jiangzhongshan Base, Tianpo Village, Mengnu Town, Longling County, Baoshan City, Yunnan Province, China (N 24°30′13″, E 99°14′58″). The experimental trees were five-year-old plants grown on a west-facing slope with a planting spacing of 4 × 6 m. Under local growing conditions, the flowering period extends from mid-February to mid-March, and commercial maturity is reached after late October. At harvest, Hass is characterized by a pyriform fruit shape and a pebbly skin texture, with an average fresh fruit weight of 250–270 g at this cultivation site, which transitions to purplish-black upon postharvest ripening.

All plant materials were collected from the same batch of healthy adult trees grown under standard orchard management practices. Six tissue types, including leaf, stamen, pistil, seed, bark, and root, were harvested simultaneously from the same trees, with three biological replicates per tissue type. For the fruit developmental series, pericarp tissue was sampled at 15 successive stages. Stages 1 through 12 were defined by days after pollination (DAP), with the first stage collected at 20 DAP and subsequent stages sampled at equal intervals thereafter. Stages 13 through 15 represented postharvest ripening, during which fruit were held at 25 °C for 0, 4, and 8 days, respectively. Each sample consisted of three biological replicates. All samples were immediately frozen in liquid nitrogen and stored at −80 °C prior to RNA extraction.

Total RNA was isolated from all samples using the RNAprep Pure Plant Kit for Polysaccharide/Polyphenol-rich samples (DP441, TIANGEN, Beijing, China). RNA quality and purity were evaluated using agarose gel electrophoresis and a NanoDrop 2000 spectrophotometer (Thermo Fisher Scientific, Waltham, MA, USA), respectively. RNA-seq libraries were constructed and sequenced on the Illumina HiSeq 2000 platform with 150 bp paired-end reads, generating a minimum of 7 Gb of raw data per sample.

### 2.2. Genome-Wide Identification and Physicochemical Characterization of PaNAC Genes

To systematically identify NAC transcription factor family members in *P. americana*, predicted protein sequences from the reference genome of Hass avocado (accession number: GCA_029852735.1) [29] were searched against PFAM profiles PF01849 (NAC) and PF02365 (NAM) using HMMER v3.0 with the TC threshold and E-value < 0.01. Candidate sequences were further validated by BLASTP (v2.10.1) against 117 *A. thaliana* NAC proteins obtained from PlantTFDB [30] using an E-value ≤ 1 × 10^−5^ and sequence identity ≥ 30%. The union of both searches was retained as the final candidate dataset, and the presence of the NAC domain was confirmed using the NCBI Batch CD-Search Tool under default parameters.

Protein physicochemical properties, including molecular weight (MW), isoelectric point (pI), and amino acid length, were computed from the complete protein sequences using the Peptides package (v2.4.6) [31] in R (v4.3.1), with pI values calculated using the EMBOSS pKa scale as implemented in the seqinr package (v4.2.36) [32]. Subcellular localization of the PaNAC proteins was predicted using the WoLF PSORT online tool (https://wolfpsort.hgc.jp/; version 0.2, accessed on 16 March 2026).

### 2.3. Gene Structure, Conserved Motif, and Phylogenetic Analysis

Conserved motifs in the PaNAC protein sequences were identified using the MEME Suite (version 5.5.5) [33]. Gene structure of the *PaNAC* genes and conserved motifs of the PaNAC proteins were visualized using TBtools software (v2.458) [34]. To investigate the evolutionary relationships of NAC transcription factors between *A. thaliana* and avocado, a total of 130 PaNAC proteins identified in this study together with 117 Arabidopsis NAC proteins were subjected to multiple sequence alignment using MAFFT (v7.490) [35] with default parameters. The resulting alignment was used for phylogenetic inference. A maximum likelihood (ML) phylogenetic tree was constructed using IQ-TREE2 (v3.0.1) [36] with the best-fit substitution model automatically selected by ModelFinder under the Bayesian Information Criterion (BIC). Branch support values were assessed using the ultrafast bootstrap approximation with 1000 replicates. The phylogenetic tree was visualized and annotated using the ggtree package (v3.8.2) [37] in R. Nodes with ultrafast bootstrap support ≥ 95% and ≥75% were indicated with filled black and red circles, respectively.

### 2.4. Chromosomal Distribution and Intra- and Inter-Genomic Synteny

Gene density was calculated per 100 kb window on each chromosome, and chromosomal distribution of *PaNAC* genes was visualized using TBtools. Intra-genomic collinearity within *P. americana* was analyzed using MCScanX [38] with default parameters. Syntenic blocks and *PaNAC* gene pairs were subsequently extracted and visualized using Circos (v0.69) [39]. Inter-genomic synteny between *P. americana* and *A. thaliana* was analyzed using MCScanX and visualized using JCVI utilities (v1.1.12) [40]. *PaNAC* gene pairs within syntenic blocks were highlighted in red for clarity.

### 2.5. Transcriptome Profiling and Differential Expression Analysis of PaNAC Genes

To further dissect expression changes associated with ripening regulation, transcriptomes were obtained from fruits at the initial ripening stage and after 4 and 8 days of treatment with 1-MCP, ethephon, and an untreated control. Three biological replicates were used for tissue and fruit developmental stage samples, while six biological replicates were used for each postharvest treatment condition.

Raw reads of RNA sequencing were quality-filtered using fastp, aligned to the reference genome of Hass avocado (accession number: GCA_029852735.1) [29] using HISAT2 (v2.2.1) [41], sorted with SAMtools (v1.10) [42], and quantified using featureCounts (v2.0.1). Read counts were normalized using TMM scaling, and differential expression analysis was conducted using DESeq2 (v1.34.0) [43] via the Trinity (v2.15.2) run_DE_analysis.pl pipeline. DEGs were defined using thresholds of |log2 fold change| ≥ 1.5 and Benjamini–Hochberg adjusted *p*-value < 0.05. For each comparison, genes were classified as upregulated (log2FC ≥ 1.5, Padj < 0.05), downregulated (log2FC ≤ −1.5, Padj < 0.05), or not significant.

### 2.6. Co-Expression Network Analysis of PaNAC Genes

To further elucidate the regulatory networks of *PaNAC* genes during fruit ripening, WGCNA was performed using TPM values derived from 15 ripening-stage samples. Co-expression modules significantly correlated with ripening traits were subsequently subjected to network construction and visualization. WGCNA was conducted using the WGCNA R package (v1.73) [44] with TPM values of all expressed genes as input. The initial expression matrix comprised 42,443 genes, from which genes with a total expression sum <= 10 across all samples were removed as lowly expressed, retaining 23,052 genes for downstream analysis. A soft-thresholding power of 14 was selected to approximate scale-free topology. Gene modules were identified using the dynamic tree-cut algorithm, and module–trait associations were evaluated using Pearson correlation. The topological overlap matrix (TOM) was computed from the signed co-expression network.

## 3. Results

### 3.1. PaNAC Genes Share Conserved Motif Composition but Exhibit Diverse Gene Structures

NAC domain-containing genes are plant-specific transcription factors representing one of the largest gene families in the model plant *Arabidopsis* and many other crop species [17]. A total of 130 *PaNAC* genes were identified in *P. americana* through a combinatorial search using HMMER profiles and BLASTP analysis (Table S1). PaNAC protein lengths ranged from 106 to 1159 AAs, molecular weights from 12 to 129 kDa, and predicted pI values from 4.10 to 10.07 (Table S2).

**Figure 1:**
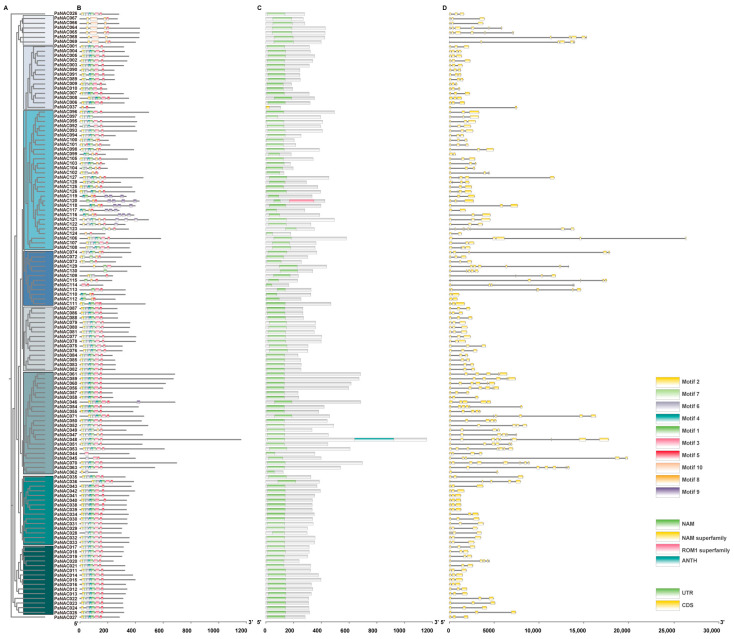
Phylogenetic relationships, motif compositions, and gene structure of *PaNAC* genes. (**A**) Multiple alignments of 130 full-length AAs of *PaNAC* genes were conducted by MAFFT, and the maximum likelihood (ML) phylogenetic tree was constructed using IQ-TREE2 with 1000 bootstrap replicates. Nodes with ultrafast bootstrap support ≥ 95% and ≥75% are indicated with filled black and red circles, respectively. The eight major phylogenetic subfamilies are highlighted with different background colors. (**B**) Schematic representation of the conserved motifs in the PaNAC proteins identified by MEME. Each motif is represented by a number in the colored box. The black lines represent the non-conserved sequences. See Figure S1 for the details of individual motifs. (**C**) NAM domain from the NCBI batch CDD of *PaNAC* genes. Domains are represented by colored boxes. (**D**) Exon/intron structures of *PaNAC* genes. Exons and introns are represented by yellow boxes and black lines, respectively. The sizes of exons and introns can be estimated using the scale at the bottom.

Phylogenetic analysis of the complete PaNAC protein sequences revealed a distinct hierarchical organization, partitioning the gene family into eight well-defined subfamilies (Figure 1A). Motif scanning using the MEME algorithm identified 20 distinct conserved sequence patterns within the PaNAC proteins (Figure 1B). Consistent with previous studies, the characteristic conserved subdomains A through E constituting the DNA-binding domain were in the N-terminal regions of most NAC proteins (Figure 1C). Motifs 2, 7, 6, 4, and 1 of PaNACs correspond to NAM subdomains A through E, respectively (Figure S1). However, some sequences displayed atypical DNA-binding domain organization, lacking one or more of the canonical five subdomains. This may reflect pseudogenization, neofunctionalization, or divergent DNA recognition mechanisms. Examination of gene architecture revealed substantial structural conservation among closely related paralogs within individual subfamilies (Figure 1D).

### 3.2. PaNAC Genes Are Unevenly Distributed Across Chromosomes

**Figure 2:**
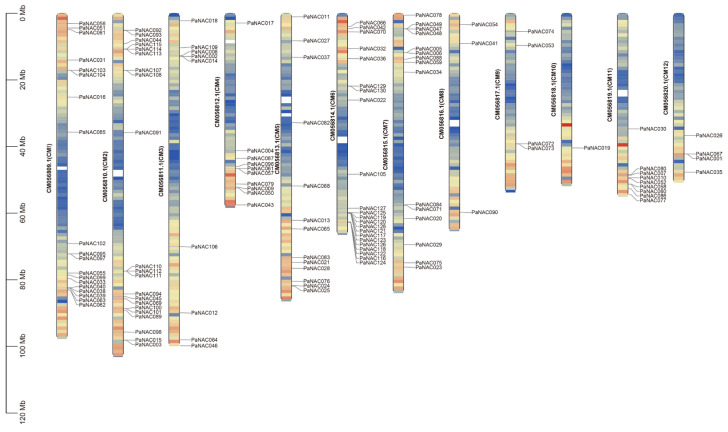
Localizations and distribution of *PaNAC* genes on *P. americana* chromosomes. The color of the pseud-chromosome indicates gene density per 100 kb.

In silico mapping of gene loci showed that 130 *PaNAC* genes were distributed across all 12 chromosomes (CM1–CM12) (Figure 2). CM6 harbored the largest number of *PaNAC* genes (*n* = 22), followed by CM2 (*n* = 21) and CM1 (*n* = 19). In contrast, only one *PaNAC* gene was identified on CM10, and three were located on CM8. Gene clustering was observed on several chromosomes, particularly those with high NAC gene density. For instance, *PaNAC125*, *PaNAC119*, *PaNAC120*, *PaNAC128*, *PaNAC121*, *PaNAC117*, *PaNAC123*, and *PaNAC126* formed one cluster on CM6, and four additional *PaNAC* genes (*PaNAC118*, *PaNAC122*, *PaNAC116*, and *PaNAC124*) were organized in a separate cluster on the same chromosome (Figure 2).

### 3.3. Conserved Synteny of PaNAC Genes

**Figure 3:**
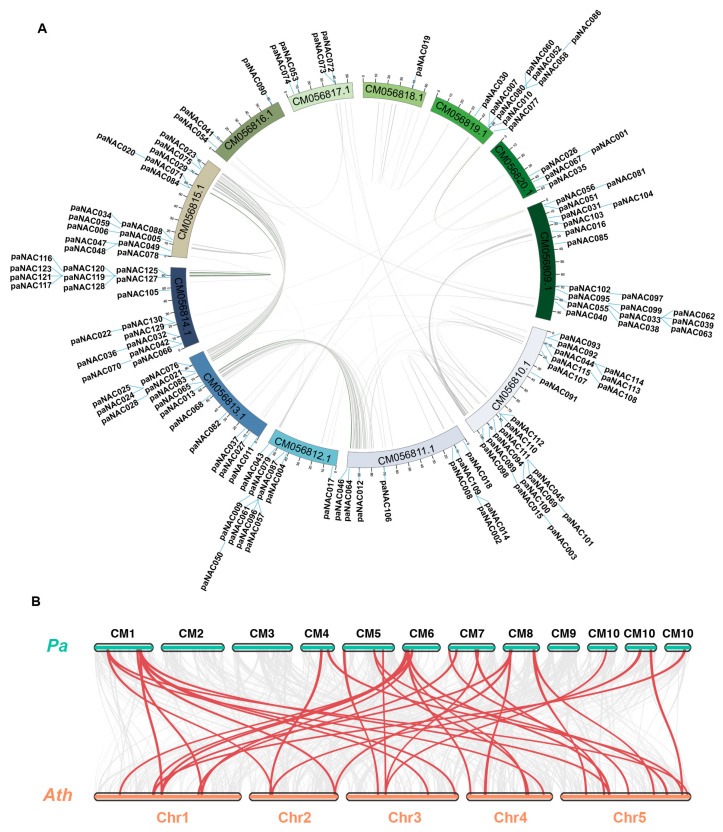
Intra- and inter-specific collinearity analysis of *PaNAC* genes. (**A**) Intraspecies collinearity analysis. The red lines represent duplication events of *PaNAC* genes and gray lines indicate all gene pairs with collinearity relationships in genome. (**B**) Synteny analysis of NAC genes in *P. americana* and *A. thaliana*. Gray lines in the background represent the collinear blocks within *P. americana* and *A. thaliana*, while the colored lines highlight the gene block have the collinear NAC gene pairs.

The conservation of gene order between chromosomes of different species (synteny) was studied. Collinearity analysis identified 11 collinear gene pairs involving 22 *PaNAC* genes within the *P. americana* genome (Figure 3A). CM3, CM5, and CM7 contained the highest numbers of duplicated gene pairs, while the remaining paralogous pairs were distributed on CM1 and CM6. To further explore the evolutionary relationships of NAC genes across species, we performed an inter-genomic collinearity analysis between *P. americana* and *A. thaliana* (Figure 3B). A total of 39 collinear gene pairs within 35 collinear blocks were identified, with 25 *PaNAC* genes, including *PaNAC005*, *PaNAC007*, and *PaNAC01*, sharing homologous relationships with *A. thaliana* counterparts. Notably, protein sequence comparison revealed that *PaNAC011* is highly homologous to *AT3G15170.1* (*AtCUC1*), suggesting that *PaNAC011* may share a similar biological function with *AtCUC1*. In the synteny map, 36 orthologous NAC gene pair blocks, highlighted in red, revealed the conserved syntenic relationships and evolutionary history of the NAC gene family between these two species.

### 3.4. PaNAC Genes Are Classified into Distinct Subfamilies Orthologous to AtNAC Groups

**Figure 4:**
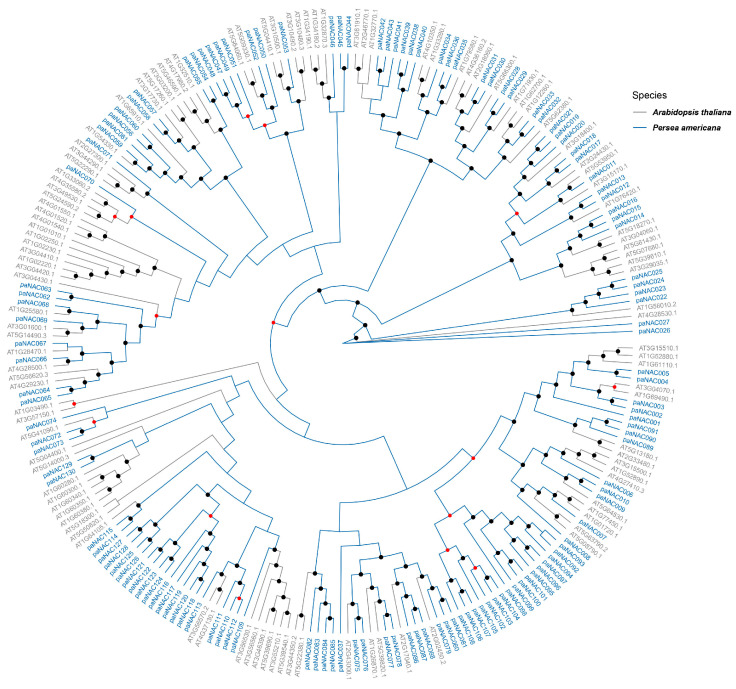
Phylogenetic tree of the *P. americana* and *A. thaliana* NAC genes. Blue branches represent *P. americana* and gray branches represent *A. thaliana*. Nodes with bootstrap support ≥ 95% and ≥75% are indicated by filled black and red circles, respectively.

To investigate the evolutionary relationships of NAC transcription factors in *P. americana*, we constructed a maximum-likelihood phylogenetic tree incorporating 130 *PaNAC* proteins and 117 well-characterized *A. thaliana* NAC members (*AtNAC*) (Figure 4). The unrooted circular phylogram was generated using IQ-TREE2 with 1000 ultrafast bootstrap replicates. Nodes with bootstrap support ≥ 95% and ≥75% are indicated by filled black and red circles, respectively, reflecting high overall topological confidence across the tree. Most *AtNAC* members from the OSNAC7, NAM, and NAC1 subfamilies were predominantly clustered in a single major branch. *PaNAC028*–*PaNAC043* were co-clustered with the OSNAC7 clade, while *PaNAC011*–*PaNAC021* were co-clustered with the NAM clade.

### 3.5. PaNAC Genes Display Tissue-Specific Expression and Ripening-Associated Co-Expression Modules

**Figure 5:**
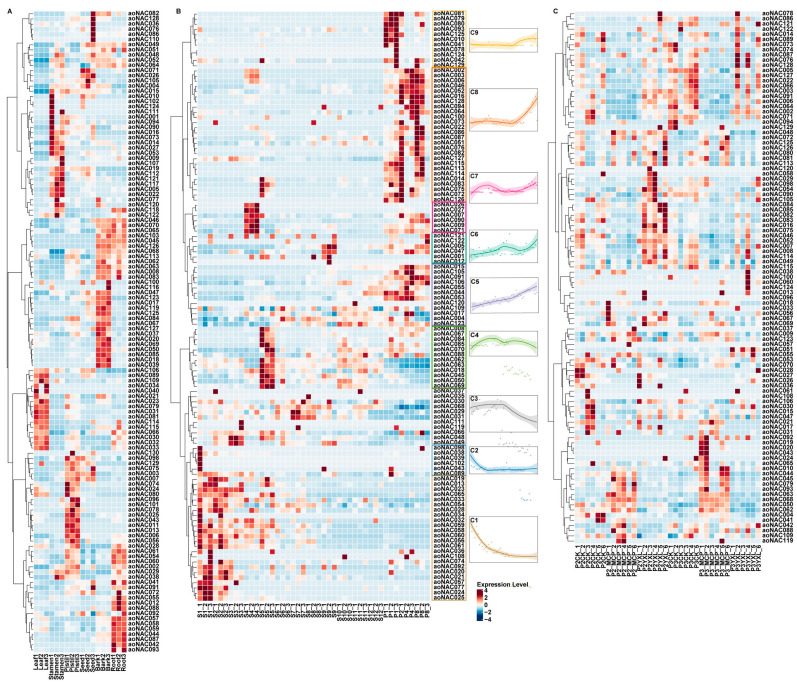
Expression profiling of *PaNAC* genes. (**A**) Heatmap showing the expression pattern of *PaNAC* genes across different tissues. Samples represent three independent biological replicates per tissue type, Leaf1–Leaf3 (leaf), Stamen1–Stamen3 (stamen), Pistil1–Pistil3 (pistil), Seed1–Seed3 (seed), Bark1–Bark3 (bark), and Root1–Root3 (root), where the numeric suffix denotes the replicate number. (**B**) Heatmap showing the expression pattern of *PaNAC* genes during fruit development and ripening. Samples are labeled as S1–S12 (fruit development stages 1–12) and P1, P2, P3 (postharvest ripening days 0, 4, and 8), each with three independent biological replicates indicated by the suffix (_1, _2, _3). The line graph represents the expression trend of each cluster. (**C**) Heatmap showing the expression pattern of *PaNAC* genes under 1-MCP and ethephon (YXL) treatment. Treatments (CK, 1-MCP, and YXL) were applied at day 0. Samples were collected at day 4 (P2CK, P2_MCP, P2YXL) and day 8 (P3CK, P3_MCP, P3YXL) after treatment, each with six independent biological replicates indicated by the suffix (_1 to _6). In all heatmaps, gene expression values are TMM-normalized (TPM), and color intensity represents row-scaled z-scores. Red indicates high relative expression and blue indicates low relative expression.

To explore the biological roles of *PaNAC* genes, we first profiled their expression across six tissue types of *P. americana*. Transcriptome sequencing of a representative sample yielded approximately 49.4 million read pairs, with 99.98% passing quality filtering (Q20 ≥ 96.96%, Q30 ≥ 92.15%) and 97.66% of reads successfully mapped to the *P. americana* reference genome, with 91.87% aligning concordantly to a unique locus. Several *PaNAC* genes showed high expression in leaves, such as *PaNAC106* and *PaNAC089* (Figure 5A), while others were predominantly expressed in stamen (e.g., *PaNAC027* and *PaNAC053*) and roots (e.g., *PaNAC042* and *PaNAC058*). To further investigate the roles of *PaNAC* genes during fruit ripening, we analyzed the expression of all 130 *PaNAC* genes across 15 fruit developmental stages. Based on k-means clustering, genes in clusters 1–3 showed high expression during pre-harvest stages of s1–s12 (Figure 5B), suggesting their involvement in regulating ripening while fruits remained on the tree. Genes in clusters 5–9 showed elevated expression during post-harvest stages p1–p3, indicating their roles in post-harvest ripening regulation.

Since regulatory genes and their targets often share similar expression patterns across development, we performed WGCNA across these 15 ripening stages. These genes were partitioned into 43 co-expression modules containing 68–3985 genes each (Figure S2). Among all modules, 68 *PaNAC* genes were assigned to 24 distinct modules, suggesting their potential roles in fruit ripening regulation. Functional enrichment analysis of these 24 NAC-containing modules revealed diverse biological processes associated with fruit ripening (Table S4). Modules enriched for hormone signaling included darkolivegreen (plant hormone signal transduction) (Table S4) and tan (abscisic acid-activated signaling pathway), while red was associated with jasmonic acid-mediated signaling and defense responses. Modules related to secondary metabolism included green (sesquiterpenoid and triterpenoid biosynthesis), royalblue (flavonoid biosynthesis), and darkturquoise (phenylpropanoid biosynthesis). Cell-wall-related processes were represented in darkolivegreen and orange (xyloglucan metabolic process). One of the largest modules, turquoise (3985 genes, 7 *PaNAC* genes), was enriched for ubiquitin-mediated proteolysis and protein processing in the endoplasmic reticulum, as well as fatty acid beta-oxidation, the latter being particularly relevant given the high lipid content and substantial fatty acid remodeling characteristic of avocado fruit ripening (Figure S3). These results indicate that *PaNAC* genes are distributed across modules associated with a range of biological processes relevant to fruit development and ripening.

**Figure 6:**
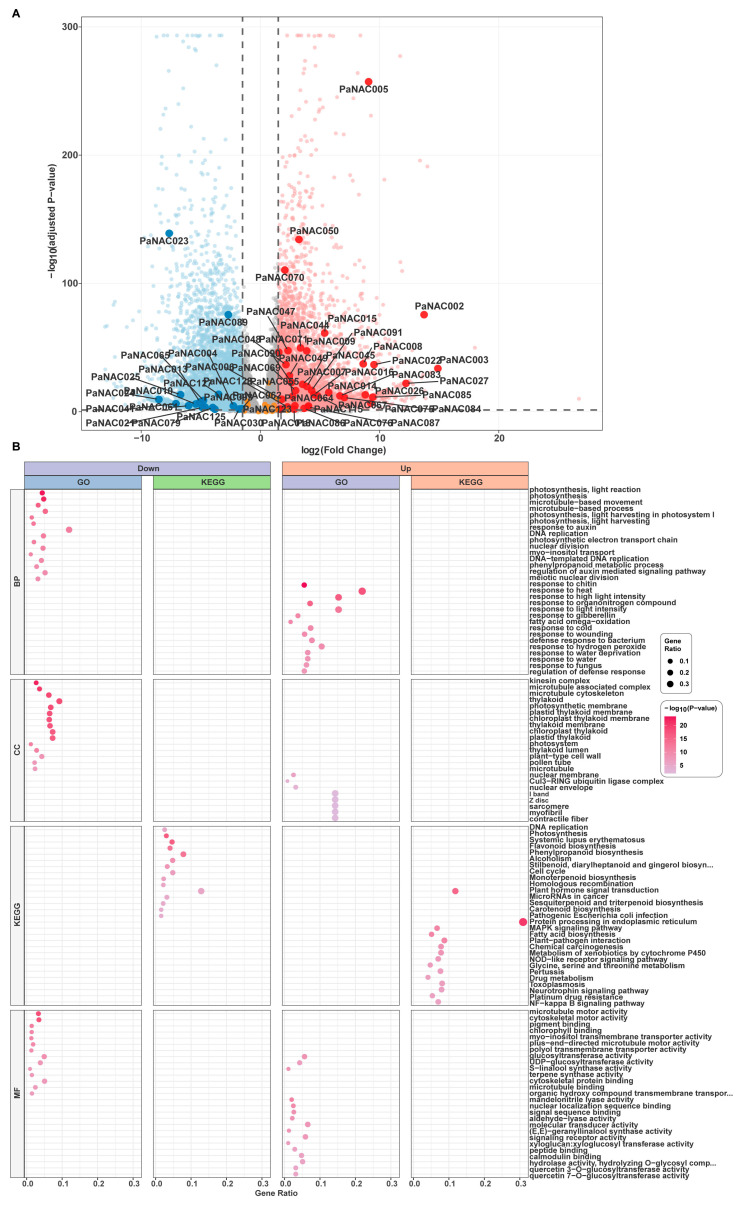
Differential expression analysis of *PaNAC* genes during avocado fruit development and postharvest ripening. (**A**) Volcano plot of differentially expressed genes (DEGs) identified across developmental and postharvest ripening stages. The *x*-axis represents log_2_(Fold Change) and the *y*-axis represents −log_10_(adjusted *p*-value). Red and blue dots indicate significantly upregulated and downregulated genes, respectively (|log_2_FC| > 1.5, adjusted *p* < 0.05). Filled circles highlight *PaNAC* genes that are differentially expressed, with gene names labeled. Vertical dashed lines indicate the log_2_FC thresholds of −1 and 1. (**B**) GO and KEGG enrichment analysis of differentially expressed genes (DEGs) across developmental and postharvest ripening stages.

To further characterize transcriptional dynamics during ripening, we identified DEGs across pairwise comparisons. A total of 48,785 DEGs were identified through 11 stages (S2–S12) compared with the S1 stage, and 2 stages (P2 and P3) compared with the P1 stage (Figure 6A). These DEGs were enriched in biological processes closely associated with fruit ripening, including plant hormone signal transduction, phenylpropanoid and flavonoid biosynthesis, carotenoid and terpenoid metabolism, fatty acid biosynthesis, cell wall modification, and defense responses to biotic and abiotic stimuli (Figure 6B, Table S5). Among these DEGs, 40 NAC genes were significantly upregulated and 30 were downregulated, with *PaNAC003* and *PaNAC002* showing the highest upregulation and *PaNAC023* and *PaNAC025* showing the strongest downregulation. These differentially expressed PaNAC genes represent strong candidates for functional roles in avocado fruit ripening.

*P. americana* is a climacteric fruit whose ripening is characterized by sharp increases in respiration rate and ethylene production, both reaching a maximum at the ripe stage of fruit development. To explore the roles of *PaNAC* genes in ethylene-mediated ripening, avocado fruits were treated with 1-MCP, a competitive inhibitor of ethylene perception, or ethephon as an ethylene donor, alongside an untreated control. A total of 9488 DEGs were identified across four comparisons of ethylene-treated versus control fruit (P2_MCP vs. P2CK, P3_MCP vs. P3CK, P2YXL vs. P2CK, and P3YXL vs. P3CK), enriched in biological processes relevant to ethylene-mediated ripening including plant hormone signal transduction, fatty acid omega-oxidation, flavonoid and secondary metabolite biosynthesis, cell wall modification, and responses to jasmonic acid and abiotic stimuli (Figure S4). Among these DEGs, 17 *PaNAC* genes were upregulated and 25 were downregulated, with *PaNAC041* showing downregulation under ethephon treatment and upregulation under 1-MCP treatment. In contrast, *PaNAC016*, *PaNAC085*, and *PaNAC086* showed elevated expression under ethylene treatment and decreased expression under 1-MCP treatment. These results suggest that *PaNAC041* may act as a negative regulator suppressed by ethylene, whereas *PaNAC016*, *PaNAC085*, and *PaNAC086* may function as ethylene-activated positive regulators during postharvest ripening.

## 4. Discussion

Avocado is a commercially important climacteric fruit whose ripening involves coordinated physiological and biochemical transitions, including softening, color change, aroma development, and a pronounced burst in respiratory activity coupled with autocatalytic ethylene production [45]. In preclimacteric avocado fruit, ethylene production is minimal due to low l-aminocyclopropane-1-carboxylic acid (ACC) synthase activity and limited ACC accumulation, with S-adenosylmethionine to ACC conversion representing the primary rate-limiting step in the ethylene biosynthesis pathway. Upon harvest, ACC synthase is de novo synthesized during the climacteric rise, triggering autocatalytic ethylene production that further induces ACC synthase activity and, once ethylene surpasses a threshold level, stimulates ethylene forming enzyme (EFE) activity to drive the full climacteric response [46]. Transcriptomic analysis of ‘Lisa’ avocado fruit pulp across preclimacteric, climacteric, and postclimacteric stages identified several ethylene-related TFs, including NAC, MYB, bHLH, and WRKY family members [47]. However, the NAC TFs in *P. americana* remain poorly characterized.

NAC transcription factors have been shown to influence ethylene biosynthesis gene expression, thereby modulating ethylene production and driving fruit ripening [7,17]. Several NAC members have been reported to positively or negatively regulate ethylene biosynthesis in tomato, peach, kiwifruit, and banana [48,49,50,51]. In the present study, we identified 130 NAC genes in avocado and found that PaNAC proteins retain the same structural organization as NAC transcription factors described in other plant species. Phylogenetic analysis with *A. thaliana* revealed that PaNAC proteins are distributed across multiple subgroups, reflecting the functional diversification of this gene family in avocado. Expression analyses support the contribution of *PaNAC* genes to fruit development and ripening. Some members were expressed across multiple tissues, such as *PaNAC049* and *PaNAC052,* suggesting roles in general developmental processes, whereas others displayed tissue-specific or fruit ripening stage-specific expression patterns. NAC genes are expressed in roots, leaves, bulbs, seeds, petals, stamen, filaments, pistils, and stems of *Lycoris radiata* [52]. Three expression patterns in general of NACs were found in the 11 successive somatic embryo developmental stages of *Liriodendron* [53]. Transcriptome analysis revealed that *PaNAC003*, *PaNAC002*, *PaNAC023*, *PaNAC025*, *PaNAC016*, *PaNAC041*, *PaNAC085*, and *PaNAC086* were selected as high-priority candidates for future functional characterization.

Functional annotation of ripening-associated and ethylene-responsive DEGs revealed GO and KEGG enrichment patterns broadly consistent with those reported in other climacteric fruits, including plant hormone signal transduction, cell wall modification, and phenylpropanoid biosynthesis, which have similarly been identified in banana and passion fruit [54,55]. A distinctive feature of the avocado DEG dataset, however, was the prominent enrichment of lipid metabolic pathways, particularly fatty acid biosynthesis, unsaturated fatty acid biosynthesis, fatty acid degradation, and α-linolenic acid metabolism, which are not typically prioritized in low-lipid climacteric fruits. Consistent with a previous transcriptome study of ‘Lisa’ avocado in which fatty acid biosynthesis, unsaturated fatty acid biosynthesis, α-linolenic acid metabolism, and fatty acid degradation were among the most enriched KEGG pathways across ripening stages [47], our analysis also identified fatty acid beta-oxidation as an enriched pathway in ripening-associated DEGs. These findings suggest that while avocado shares a conserved transcriptional framework for ripening regulation with other climacteric fruits, the prominent enrichment of fatty acid metabolic pathways represents a recurrent and likely species-specific signature reflecting the unique lipid-rich nature of avocado mesocarp. Future studies should prioritize qRT-PCR validation of ripening-associated candidate genes including the identified *PaNAC* members, promoter-binding assays to determine their direct transcriptional targets, and functional characterization through transgenic avocado or heterologous expression systems to establish their causal roles in postharvest ripening regulation.

## 5. Conclusions

This study presents the systematic genome-wide characterization of the NAC transcription factor family in *P. americana*, providing an integrated resource that links gene structure, phylogenetic relationships, chromosomal distribution, and transcriptional dynamics across fruit development, postharvest ripening, and hormone treatment. These findings establish a foundation for future functional investigation of *PaNAC* genes and their potential applications in avocado crop improvement.

## Data Availability

The original contributions presented in this study are included in the article/Supplementary Materials. Further inquiries can be directed to the corresponding authors.

## Supplementary Materials

The following supporting information can be downloaded at: https://www.mdpi.com/article/10.3390/genes17060706/s1, Figure S1: Sequence logos of conserved motifs identified in the PaNAC proteins; Figure S2: WGCNA co-expression module identification across avocado fruit ripening stages; Figure S3: GO and KEGG enrichment analysis of genes in the turquoise and darkolivegreen co-expression modules identified by WGCNA; Figure S4: GO and KEGG enrichment analysis of differentially expressed genes (DEGs) in response to ethephon and 1-MCP treatments during postharvest ripening; Table S1: Summary of *PaNAC* genes identified through NCBI CD-search with NAM domain confirmation; Table S2: Physicochemical properties of 130 PaNAC proteins. Table S3: Differential expression significance of *PaNAC* genes between sample groups as determined by DESeq2 (adjusted *p* < 0.05). Table S4: GO and KEGG enrichment analysis of the 24 co-expression modules containing *PaNAC* genes. Table S5: GO and KEGG enrichment analysis of differentially expressed genes (DEGs) identified across developmental (S2–S12 vs. S1) and postharvest (P2–P3 vs. P1) comparisons.
