# Supplementary material for: Genome-Wide Identification and Characterization of NAC Transcription Factors in Avocado (Persea americana): Expression Analysis During Fruit Development
Source: Genes (Basel). 2026 Jun 18;17(6):706. doi: 10.3390/genes17060706 (PMC13300513; doi:10.3390/genes17060706)

SUPPLEMENTARY MATERIALS

Figure S1. Sequence logos of conserved motifs identified in the PaNAC proteins. Motif scanning was performed using the MEME algorithm. In each sequence logo, the overall height of the amino acid stack indicates the degree of sequence conservation at that position (measured in bits), while the height of individual letters represents the relative frequency of that specific amino acid. (A–J) correspond to Motifs 1–10, respectively.

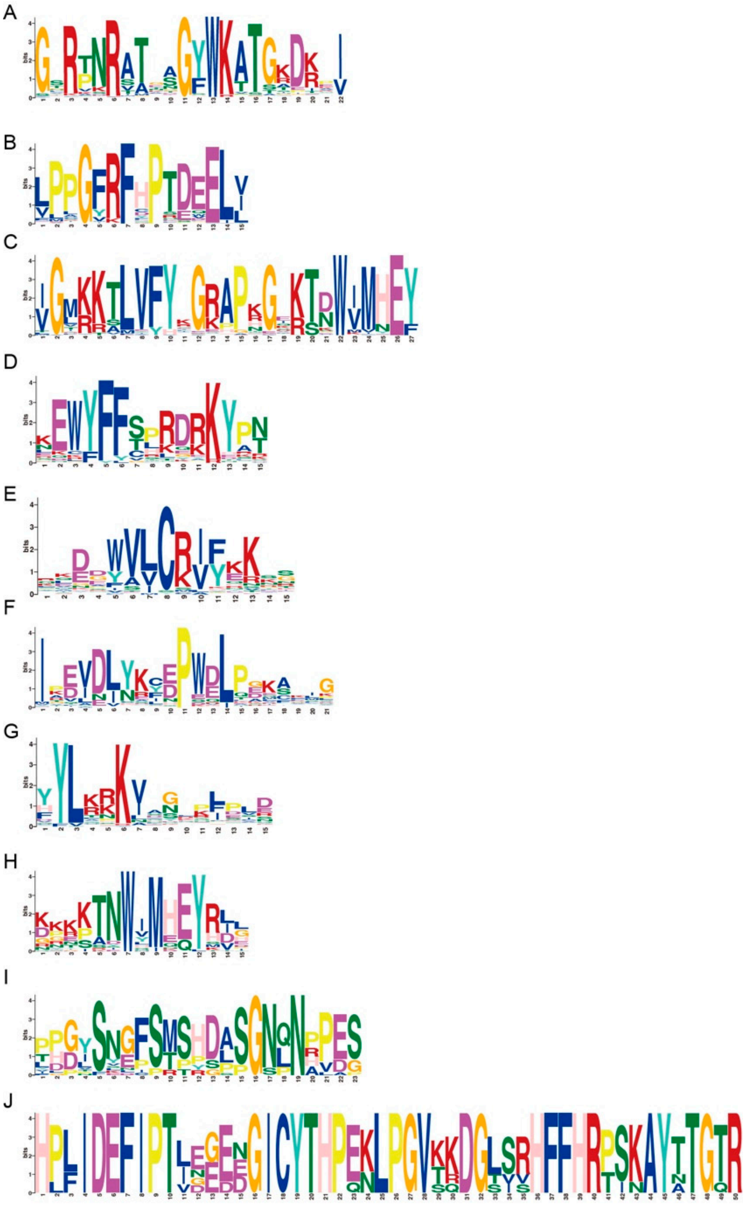

Figure S2. WGCNA co-expression module identification across avocado fruit ripening stages. Hierarchical clustering dendrogram of 23,052 genes based on topological overlap matrix (TOM)-derived dissimilarity. Each leaf represents one gene. The color band below the dendrogram indicates module assignment, with each color representing a distinct co-expression module. Genes that did not cluster into any module are assigned to the grey module.

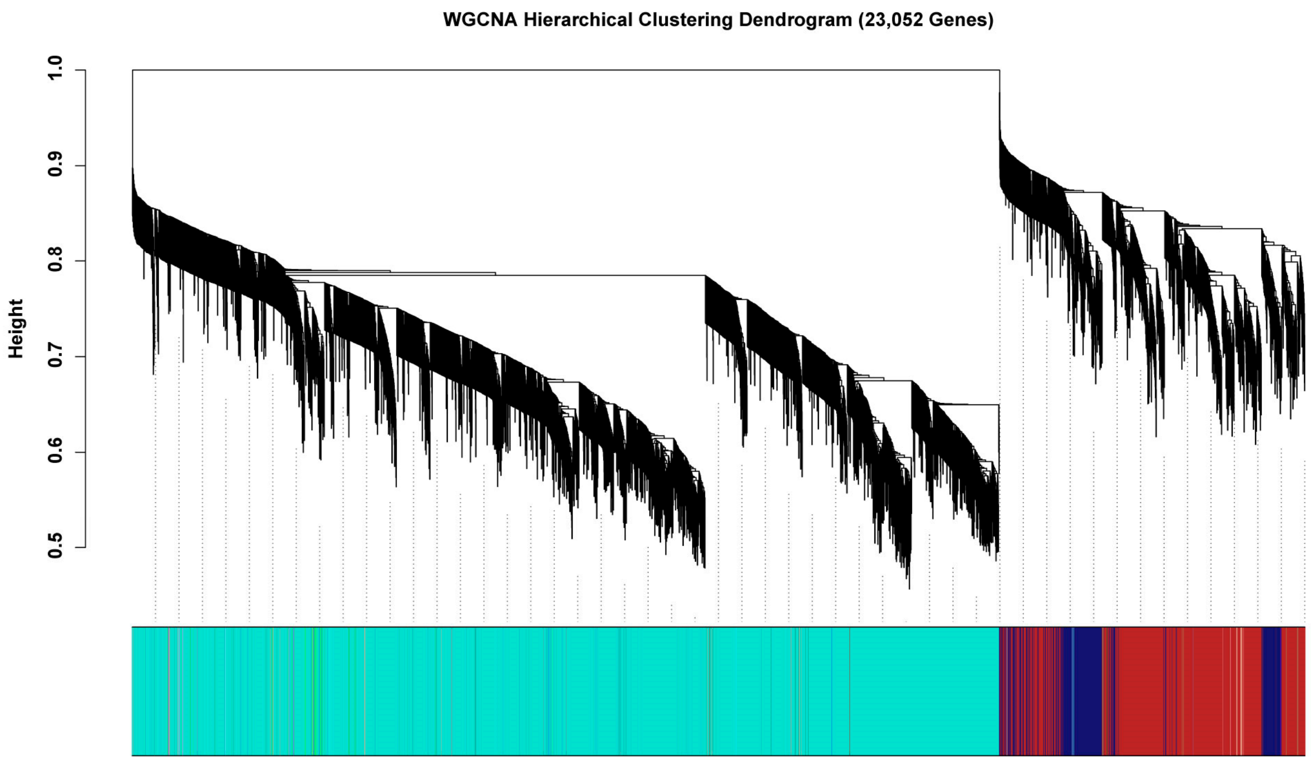

Figure S3. GO and KEGG enrichment analysis of genes in the turquoise and darkolivegreen co-expression modules identified by WGCNA.

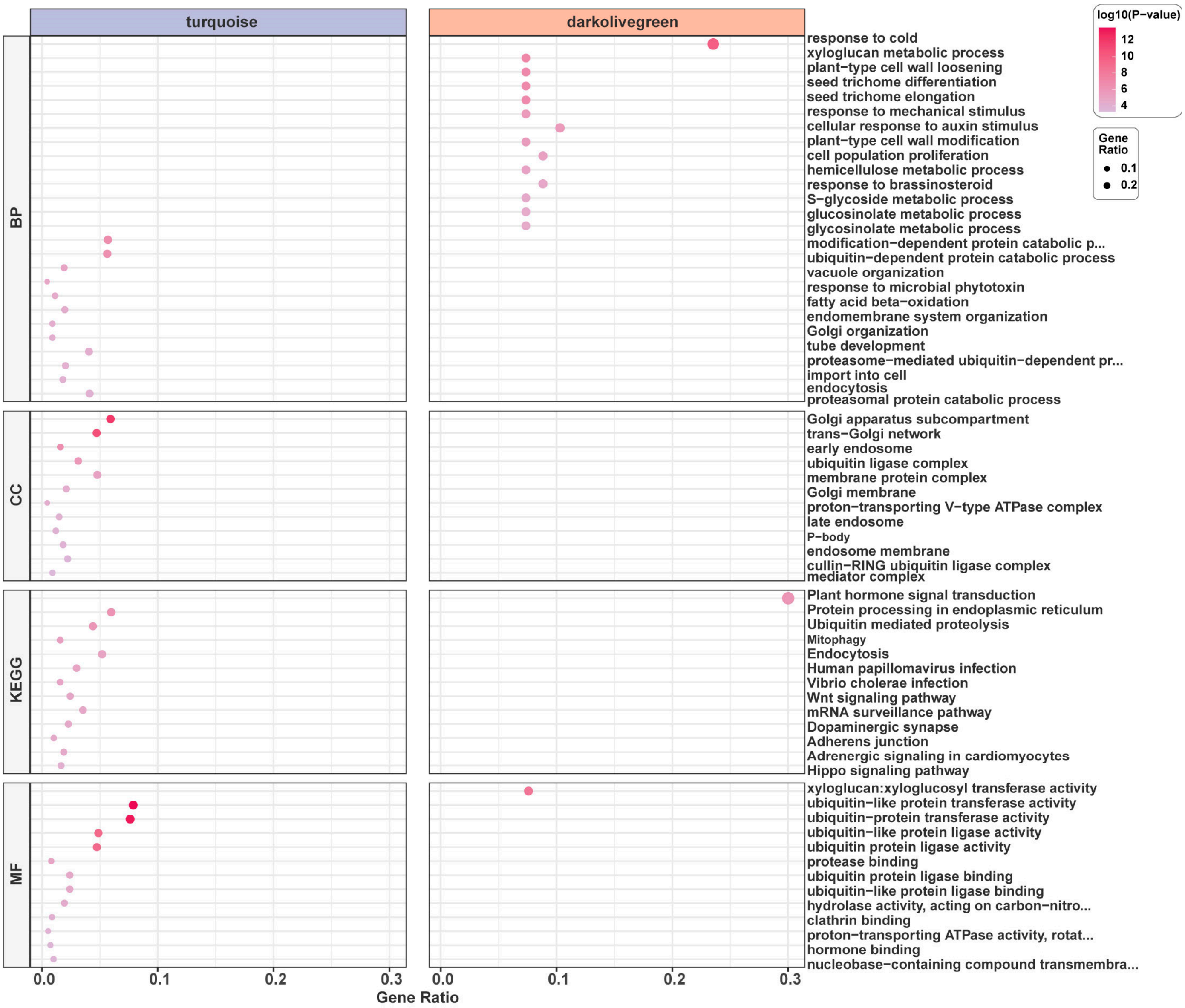

Figure S4. GO and KEGG enrichment analysis of differentially expressed genes (DEGs) in response to ethephon and 1-MCP treatments during postharvest ripening.

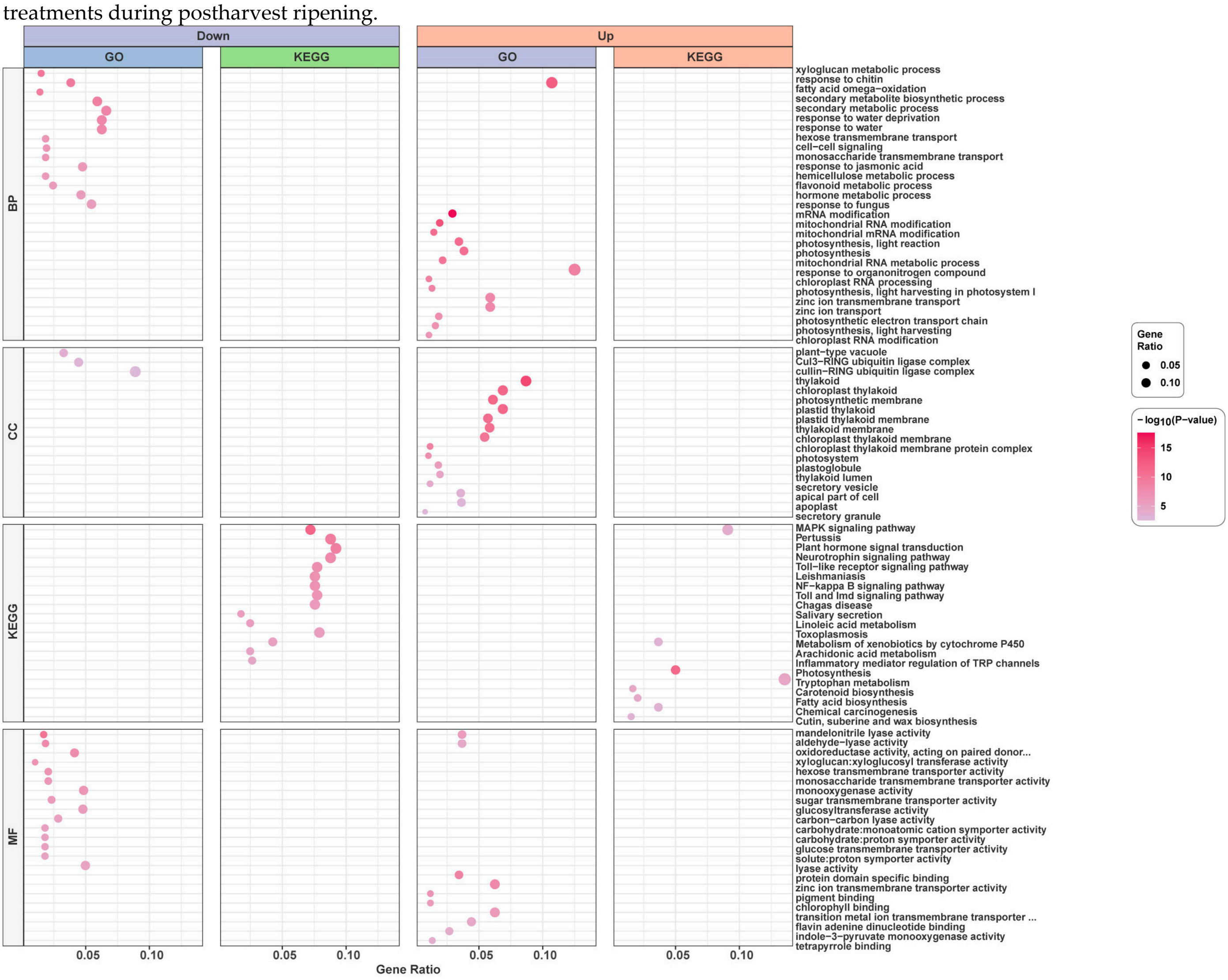

Supplement: Supplementary file 1 [file genes-17-00706-s001.zip › aonac_supplementary_figures.pdf]
